# Diagnostic Accuracy of GPT-4 With Vision in Neuroradiology Board-Style Exam Questions: Cross-Sectional Case-Based Study

**DOI:** 10.2196/69708

**Published:** 2026-04-30

**Authors:** Tom T Sussan, Rebekah R Brawley, Joshua Eckroth, James E Mossell, Tao Weitao

**Affiliations:** 1 Lake Erie College of Medicine, 5000 Lakewood Ranch Blvd., Bradenton, FL, 34211, United States, 1 9417825761; 2 Department of Computer Science, Stetson University, Deland, FL, United States; 3 College of Medicine, University of Central Florida, Orlando, FL, United States; 4 Department of Radiology, University of Florida College of Medicine, Gainesville, FL, United States

**Keywords:** neuroradiology, GPT-4 with Vision, GPT-4V, artificial intelligence, diagnostic accuracy, multimodal AI, medical imaging, clinical decision-making, text-image integration, board exam questions, RSNA Case Collection

## Abstract

**Background:**

Multimodal artificial intelligence systems combining text and image analysis represent a paradigm shift in clinical decision support. While GPT-4 with Vision (GPT-4V) has shown promise in medical imaging interpretation, existing studies report inconsistent performance (16%‐80% accuracy) across radiological subspecialties. Critical knowledge gaps persist regarding GPT-4V’s capability to integrate clinical history with imaging findings in complex neuroradiology scenarios, and fundamental questions remain about whether the model appropriately balances visual and textual information sources when formulating diagnoses. Furthermore, documented artificial intelligence hallucination rates of 35.5% to 63% in radiology applications raise urgent safety concerns, yet the relationship between modality utilization patterns and diagnostic accuracy remains unexplored.

**Objective:**

This study aims to evaluate GPT-4V’s diagnostic accuracy on expert-validated neuroradiology board-style examination questions and to examine the model’s self-reported reliance on imaging versus clinical text data when making diagnostic decisions. A secondary objective was to examine whether self-characterized modality utilization patterns differed systematically between correct and incorrect diagnoses, potentially identifying specific failure modes requiring targeted mitigation strategies.

**Methods:**

This cross-sectional study evaluated GPT-4V using 29 neuroradiology cases from the RSNA (Radiological Society of North America) Case Collection, covering adult brain and central nervous system pathologies imaged via computed tomography or magnetic resonance imaging. The cases were authored by board-certified radiologists. GPT-4V was accessed via ChatGPT Plus (July 2024) with standardized prompts selecting 1 answer from 4 options, providing diagnostic rationale, and quantifying the percentage contributions of image versus text data. Binary scoring assessed diagnostic performance (correct=1, incorrect=0). Statistical analysis included Wilson score CIs, a binomial test comparing accuracy to chance, and a 2-tailed *t* test comparing self-reported modality reliance between correct and incorrect diagnoses (α=.05, Cohen *d* calculated).

**Results:**

GPT-4V correctly diagnosed 22 of 29 cases (76% accuracy, 95% CI 57.9%-87.8%), significantly exceeding the chance performance of 25% (*z*=6.33; *P*<*.*001). The model self-reported mean contributions of 66.1% from imaging (95% CI 63.5%‐68.8%) and 33.9% from text (95% CI 31.2%‐36.5%). Correct diagnoses (n=22) showed significantly lower self-reported image reliance (62.8%, 95% CI 61.3%‐64.3%) compared to incorrect diagnoses (n=7; 76.7%, 95% CI 73.5%‐80.0%), with a mean difference of 13.9 percentage points (95% CI 10.6‐17.3; *P*<.001; Cohen *d*=4.08, 95% CI 2.73‐5.43). All 7 incorrect diagnoses demonstrated image-dominant attribution ≥70% (Fisher exact test *P*<*.*001), suggesting that excessive visual reliance may indicate diagnostic risk.

**Conclusions:**

The 76% accuracy substantially exceeds prior GPT-4V radiology studies (43%), demonstrating that focused domain application with structured prompting enhances performance. Incorrect diagnoses are associated with higher self-reported visual reliance, suggesting a potential failure mode warranting experimental validation. This pattern identifies a potentially actionable signal for quality assurance systems. Clinical deployment should remain restricted to supervised educational applications with mandatory radiologist oversight until balanced context-aware integration is validated.

## Introduction

### Background

The advent of multimodal artificial intelligence (AI) systems represents a transformative shift in medical diagnostics, particularly in radiology, where clinical decision-making requires integrated analysis of imaging findings and clinical context. Multimodal AI models combine diverse data modalities, such as imaging, text, structured records, and physiological signals, into unified analytical frameworks [1-3]. Recent advancements in transformer architectures and foundation models have enabled unprecedented capabilities in processing heterogeneous medical data [4-6], with AI adoption in radiology accelerating rapidly in recent years [7].

OpenAI’s GPT-4 with Vision (GPT-4V), released in 2023, exemplifies this multimodal paradigm by enabling the simultaneous interpretation of text and images. Large language models (LLMs) have demonstrated utility in radiology report generation, board exam preparation, and clinical decision support [8-11], with studies reporting significant improvements in efficiency and consistency [7]. However, the addition of visual integration has yielded contradictory performance patterns across radiological subspecialties, raising fundamental questions about how these models process and integrate information from different modalities.

### Current Evidence and Critical Knowledge Gaps

Empirical evaluations reveal substantial heterogeneity in GPT-4V diagnostic accuracy. Huppertz et al [12] demonstrated that diagnostic accuracy improved from 8.3% with images alone to 29.1% with contextualized prompts, though the model exhibited pronounced context bias and frequent fabricated findings, with similar concerns documented in other multimodal evaluations [13]. Studies report GPT-4V accuracy ranging from 16% to 49% in challenging radiology cases (characterized by rare pathologies, subtle findings, or complex differentials), consistently below trained radiologists’ performance [14-16]. Albaqshi et al [17] found that among 6 LLMs evaluated on 56 neuroradiology cases, Claude 3.5 achieved the highest accuracy (80.4%), with LLMs performing comparably to first-year fellows while showing high consistency across repeated queries. Systematic reviews confirm variable results (16%‐80% accuracy) depending on case difficulty, prompt engineering, and domain specificity [18,19].

Fundamental questions persist about how GPT-4V integrates visual and textual information. Multiple studies document limited visual interpretation capabilities: Schramm et al [20] identified textual descriptions as the strongest contributor to performance, while Albaqshi et al [17] demonstrated that image-only accuracy plummeted to 21.5% to 63.1% compared to 62.5% to 76.8% with combined inputs. Conversely, some studies show GPT-4V’s superiority over text-only approaches [21], while others report text-only models outperforming multimodal implementations [16,22,23]. These findings suggest a critical paradox: adding visual capabilities may not enhance but can potentially degrade diagnostic performance when multimodal integration is suboptimal.

A critical barrier to clinical deployment is AI hallucinations, the plausible but incorrect information that appears factually grounded [7,24-26]. LLM hallucinations in medical contexts remain a critical concern [7], manifesting as fabricated findings or misidentified modalities [12,26,27]. Jin et al [24] documented “hidden flaws behind expert-level accuracy,” revealing systematic errors obscured by superficially correct outputs. Current literature lacks systematic investigation of whether diagnostic failures correlate with specific modality utilization patterns. Understanding these patterns is essential for safe deployment, as systematic overreliance on either modality could lead to predictable failure modes requiring targeted mitigation.

Current multimodal foundation models exhibit limitations precluding autonomous diagnostic use: inconsistent results across identical inputs, tendency toward confabulation [26,27], sensitivity to prompt engineering [28], lack of transparency [12,25], and variable performance across modalities and anatomical regions [12,29]. Recent position statements emphasize that AI integration must prioritize human-AI collaboration frameworks, transparent uncertainty quantification, and mandatory expert oversight [7,30]. Despite growing literature on diagnostic accuracy, critical gaps remain regarding (1) how multimodal AI characterizes its reliance on visual versus textual inputs, (2) whether modality attribution patterns differ between correct and incorrect diagnoses, and (3) whether self-reported information utilization reflects actual processing versus post hoc rationalization [17].

### Study Objectives

This study addressed two objectives: (1) to evaluate GPT-4V’s diagnostic accuracy on expert-validated neuroradiology board-style questions, providing benchmark performance data under standardized conditions and (2) as an exploratory analysis to document GPT-4V’s self-reported reliance on imaging versus clinical text and examine whether self-characterized modality utilization patterns differ between correct and incorrect diagnoses. We acknowledge that determining whether these self-assessments reflect actual information processing versus post hoc rationalization requires rigorous experimental validation through controlled text-only and image-only conditions.

## Methods

### Study Design and Data Source

This cross-sectional study, reported according to JARS-Quant guidelines [31], evaluated GPT-4V’s diagnostic accuracy using 29 neuroradiology cases from the RSNA (Radiological Society of North America) Case Collection. The cases included adult brain and central nervous system pathologies imaged via computed tomography (CT) or magnetic resonance imaging (MRI; Table S1 in Multimedia Appendix 1, Figure S5.1 and Table S2.1 in Multimedia Appendix 2, and Table S6.1 in Multimedia Appendix 3). Each case included a clinical vignette and diagnostic-quality imaging studies (Figures S2 in Multimedia Appendix 4 and Figure S3 in Multimedia Appendix 5, respectively). The inclusion criteria required expert-verified diagnoses in multiple-choice format; the cases were authored by board-certified radiologists and underwent editorial review following established quality standards for educational radiology assessments [32], analogous to standardized board examination validation. Cases were excluded if they involved pediatric patients, lacked diagnostic images, or had no definitive correct answer.

While the RSNA Case Collection’s restricted membership access reduces the likelihood of training data contamination, we acknowledge that with closed-source models, data leakage cannot be definitively ruled out and could artificially inflate performance estimates. All case materials were deidentified and used with permission. The cases were accessed in July 2024.

### Assessment and Scoring Methodology

Complete prompt structure, standardized instructions, and example responses are documented in Multimedia Appendix 4 (Parts A-E), ensuring reproducibility. Binary scoring evaluated diagnostic performance: correct (score=1) if the model’s answer matched the peer-reviewed correct diagnosis from RSNA documentation; incorrect (score=0) otherwise (Table S6.1 in Multimedia Appendix 3 and Table S7.1 and Section 8.1 in Multimedia Appendix 6). All cases underwent peer review and editorial vetting by the RSNA’s editorial board prior to publication (Multimedia Appendix 5). No partial credit was given. Overall accuracy was calculated as percentage correct out of 29 cases. As exploratory measures, we recorded self-assessed percentage influence of image versus text for each case, emphasizing that these represent subjective self-reports rather than validated measurements of actual information contribution (Table S7.2 and Section 8.1 in Multimedia Appendix 6).

### Ethical Considerations

This study did not constitute human subjects research as defined by US Department of Health and Human Services regulations at 45 CFR 46.102(e) and (l) [33]. The study involved secondary analysis of fully deidentified educational case materials from the RSNA Case Collection, accessed through authorized membership. No living individuals were contacted, and no identifiable private information was obtained, used, or generated. The RSNA Case Collection requires authors to remove all patient identifiers prior to submission (Multimedia Appendix 5). All figures and multimedia appendices in this study contain only fully deidentified radiological images and clinical information from the RSNA Case Collection, with no possibility of individual identification.

### Missing Data Analysis

All 29 cases included in the analysis had complete data for the primary outcome (diagnostic accuracy; Table S5.1 in Multimedia Appendix 7). Each case successfully elicited a diagnostic response from GPT-4V, with the model selecting 1 of 4 answer options in all instances. For the exploratory modality attribution measures, the model provided self-reported percentage contributions (image vs text) for all 29 cases, resulting in zero missing data for both primary and exploratory outcome measures (Figure S5.1 in Multimedia Appendix 7 and Part C in Multimedia Appendix 4). Therefore, no imputation procedures or missing data analyses were necessary. The completeness of data reflects the controlled nature of the study design, where GPT-4V was systematically prompted to provide both diagnostic answers and modality attribution percentages for each case. One trial per case was conducted with standardized prompts designed to elicit complete responses. The single-trial design means that response variability across multiple trials was not assessed. GPT-4V’s temperature setting and stochastic sampling could produce different responses on repeated trials; this variability is addressed in the *Limitations* section. The study protocol specified that any case with incomplete model responses would be excluded and reported as a protocol deviation. This scenario did not occur.

### Statistical Considerations

The sample size (N=29) was determined by available cases meeting the inclusion criteria (Tables S3.1 and S3.2 in Multimedia Appendix 8 document post hoc power >99.9% for primary analysis, 97% for exploratory analysis, and ±16.3 percentage point margin of error). Statistical significance was assessed using a 2-sided alpha level of .05 for all hypothesis tests. Statistical analysis was primarily descriptive (Tables S6.1-S6.2 in Multimedia Appendix 3). Statistical analysis used a 2-sample 2-tailed *t* test with standard error–based CIs for continuous variables; Wilson score method was applied separately for the diagnostic accuracy proportion (Table S4.2 in Multimedia Appendix 9 presents complete 2-tailed *t* test results: *t*_27_=9.40; *P<.*001, Cohen *d*=4.08; Tables S5.3-S5.5 in Multimedia Appendix 7 verify assumptions, including normality and equal variances, and provide comprehensive descriptive statistics). We compared the findings qualitatively to previous studies [20-23,30].

A 1-sample binomial test assessed whether diagnostic accuracy exceeded random guessing (25% for 4-option questions, *z*=6.33, *P<.*001; Table S4.1 in Multimedia Appendix 9). CIs for proportions were calculated using the Wilson score method. For modality weighting, 95% CIs were calculated using the *t* distribution. A 2-sample 2-tailed *t* test compared self-reported image reliance between correct and incorrect cases. Effect sizes (Cohen *d*) with 95% CIs are reported to allow readers to interpret clinical and statistical significance (Table S4.3 in Multimedia Appendix 9 shows all incorrect diagnoses demonstrated image-dominant attribution ≥70%, Fisher exact test *P<.*001; Table S5.2 in Multimedia Appendix 7 confirms no statistical outliers; Tables S7.1 and S7.2 and Section S8.2 in Multimedia Appendix 6 document variable definitions and derived measures). Complete statistical methods are detailed in Multimedia Appendix 9.

**Figure 1.**
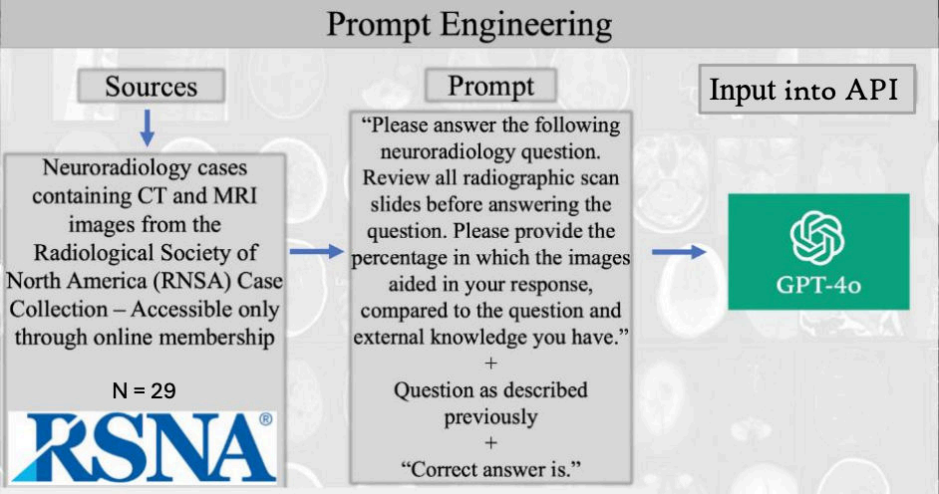
Prompt engineering workflow for GPT-4 with Vision (GPT-4V) evaluation in neuroradiology board-style questions: a cross-sectional study of 29 adult brain and central nervous system pathologies from the RSNA Case Collection (July 2024). API: application programming interface; CT: computed tomography; MRI: magnetic resonance imaging.

Figure 1 is the systematic methodology for evaluating GPT-4V diagnostic performance on 29 neuroradiology cases from the RSNA Case Collection. The workflow includes (1) data sources—cases containing CT or MRI scans of adult brain and central nervous system pathologies, clinical vignettes, and peer-reviewed multiple-choice questions; (2) standardized prompt structure—a consistent template instructing GPT-4V to review all radiographic imaging, select 1 diagnostic answer from 4 options, provide diagnostic rationale, and quantify the percentage contribution of visual versus textual information to its diagnostic decision (exploratory outcome measure); and (3) ChatGPT Plus implementation—prompt delivery via ChatGPT Plus web interface. This structured methodology ensures standardized evaluation while systematically capturing the model’s self-reported reliance on visual versus textual information sources. One trial per case was conducted without iterative prompting to simulate real-world clinical conditions where single diagnostic assessments are typical.

**Figure 2.**
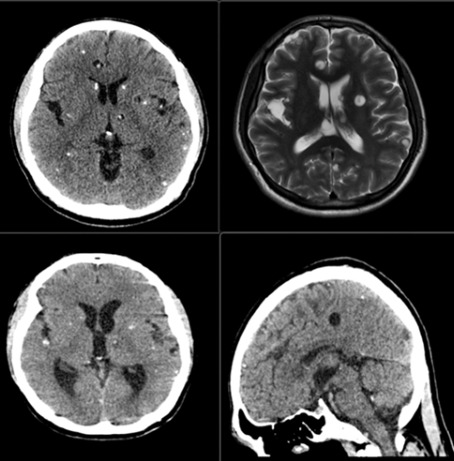
Representative infectious disease neuroradiology case from the RSNA Case Collection demonstrating clinical context integration in GPT-4 with Vision (GPT-4V) multimodal diagnostic decision-making.

Figure 2 is a representative infectious disease case (Table S6.1 in Multimedia Appendix 3, Case #14: Neurocysticercosis) from the RSNA Case Collection, 1 of 29 adult brain and central nervous system pathology cases (Figure S5.1 in Multimedia Appendix 2, Table S6.1 in Multimedia Appendix 3) used in this July 2024 cross-sectional evaluation (Figure S5.1 in Multimedia Appendix 2) of GPT-4V diagnostic accuracy. This case features a 32-year-old male presenting with first-time seizure (complete case vignette in Multimedia Appendix 5) and includes (1) a clinical vignette containing patient demographics, symptoms, and history (textual information) and (2) diagnostic-quality neuroimaging studies in PNG format (image format not documented in appendices) obtained via CT or MRI (visual information; Multimedia Appendix 5 and Figure S5.1 in Multimedia Appendix 2). GPT-4V was required to integrate both clinical context and imaging findings (Part A: standardized prompt structure in Multimedia Appendix 4) to select the correct diagnosis from 4 multiple-choice options (Part A in Multimedia Appendix 4 and Answer Choices A-D in Multimedia Appendix 5), with self-reported percentage contributions from each modality recorded as an exploratory measure (Parts A-B in Multimedia Appendix 4; Table S6.1 in Multimedia Appendix 3 confirms 65% image, 35% text attribution for this case).

**Figure 3.**
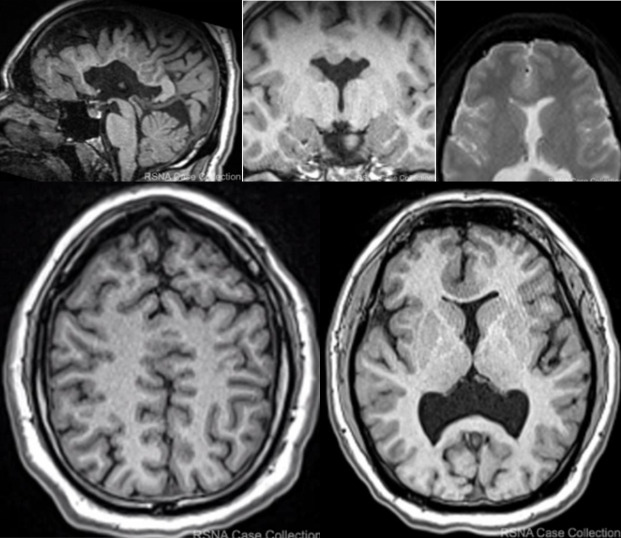
Representative developmental brain malformation case highlighting complex multimodal integration requirements for accurate artificial intelligence (AI) diagnosis.

Figure 3 is a complex neuroradiology case (Table S6.1 in Multimedia Appendix 3, Case #19: Syntelencephaly) from this July 2024 cross-sectional study (Figure S5.1 in Multimedia Appendix 2) evaluating GPT-4V’s diagnostic performance on 29 adult brain and central nervous system pathology cases from the RSNA Case Collection. This case involves a 31-year-old male patient with developmental brain abnormalities and seizures (Multimedia Appendix 5: complete case presentation with clinical vignette, developmental history). The case structure provided to GPT-4V included (1) clinical vignette describing patient demographics, symptoms (seizures), developmental history, and relevant neurological findings (textual data) and (2) complete series of diagnostic-quality neuroimaging studies obtained via CT or MRI in standardized PNG format (image format not documented); visual data (Part A in Multimedia Appendix 4 and Multimedia Appendix 5). This case exemplifies challenging diagnostic scenarios where developmental malformations present subtle imaging findings requiring expert-level integration of both clinical context and radiological interpretation (Table S6.1 in Multimedia Appendix 3 confirms correct diagnosis; Table S2.1 in Multimedia Appendix 2 shows Developmental category: 100% accuracy).

The mean self-reported modality contributions across diagnostic outcomes for 29 neuroradiology cases from the RSNA Case Collection are presented. The data are shown for all cases (N=29), correct diagnoses (n=22), and incorrect diagnoses (n=7). Image contributions are normalized to 1.0 (blue bars) to enable comparison across categories; text contributions appear as ratios (purple bars). Error bars represent 95% CIs for text:image ratios. Incorrect diagnoses showed significantly higher self-reported image reliance (76.7%) compared to correct diagnoses (62.8%), with a mean difference of 13.9 percentage points (*P<.*001; Cohen *d*=4.08; Tables S4.2-S4.3 in Multimedia Appendix 9).

## Results

### Overview of Primary and Exploratory Findings

Our primary finding is that GPT-4V achieved 76% diagnostic accuracy (22 out of 29 correct diagnoses) on expert-validated neuroradiology cases, significantly exceeding chance performance. Secondary exploratory findings regarding self-reported modality utilization should be interpreted cautiously, as they represent the model’s characterization of its process rather than validated measurements of actual information use.

### Diagnostic Performance

GPT-4V correctly diagnosed 22 out of 29 neuroradiology cases, yielding 76% accuracy (95% CI 57.9%‐87.8% by Wilson method), significantly above the 25% expected by chance (*z*=6.33; *P<.*001; Table S1 in Multimedia Appendix 1, Table S4.1 in Multimedia Appendix 9, and Table S6.1 in Multimedia Appendix 3). This exceeds the 43% accuracy (31/72 cases) reported by Mukherjee et al [34] for GPT-4V on RSNA “Case of the Day” challenges, suggesting that within focused domains under structured prompting, GPT-4V’s performance can be enhanced. The multiple-choice format (Part A in Multimedia Appendix 4 and Multimedia Appendix 5) may have aided performance by providing plausible options rather than requiring open-ended diagnosis generation. However, we acknowledge that data leakage cannot be definitively ruled out with closed-source models, and any training data contamination could have contributed to this performance.

### Multimodal Data Integration Patterns

**Figure 4.**
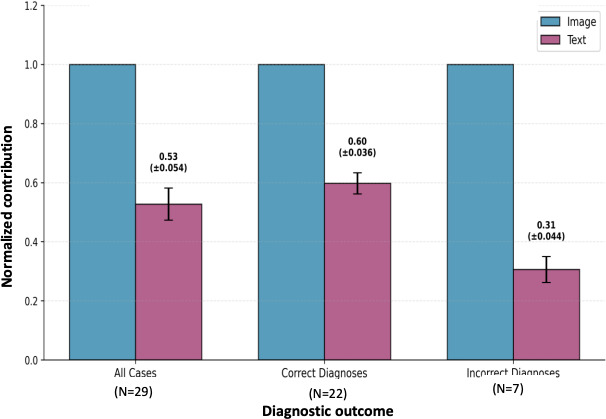
Self-reported image and text contributions by diagnostic outcome in GPT-4V neuroradiology evaluation.

As an exploratory analysis, we examined GPT-4V’s self-reported percentage contributions of visual versus textual information to diagnostic decisions. The model reported that image data contributed 66% (95% CI 63.5%‐68.8%) and textual data 34% (95% CI 31.2%‐36.5%) on average (Table S1 in Multimedia Appendix 1 and Table S5.5 in Multimedia Appendix 7). The overall text:image ratio was 0.53; however, this ratio varied by diagnostic outcome: correct diagnoses showed 0.60 versus incorrect diagnoses at 0.31 (Figure 4; Table S4.2 in Multimedia Appendix 9), suggesting that errors may be associated with self-characterized overreliance on imaging (Table S4.3 in Multimedia Appendix 9 shows that 100% of incorrect diagnoses had image-dominant attribution ≥70%, Fisher exact *P<.*001; Table S6.1 in Multimedia Appendix 3 provides case-level data). Whether this reflects actual information processing or post hoc rationalization requires rigorous experimental validation through controlled text-only and image-only conditions (Section 8.1 in Multimedia Appendix 6 documents interpretation limitations and validation requirements).

### Comparative Analysis

Our 76% accuracy (95% CI 57.9%-87.8%; Table S4.1 in Multimedia Appendix 9 and Table S1 in Multimedia Appendix 1) substantially exceeds the 43% from Mukherjee et al [34], who found that GPT-4V performed worse on imaging-dependent cases (39%) than on text-only inputs (50%), suggesting heavy reliance on textual information. Our focused neuroradiology approach (Table S6.1 in Multimedia Appendix 3 and Table S2.1 in Multimedia Appendix 2) with multiple-choice format (Part A in Multimedia Appendix 4 and Multimedia Appendix 5) was associated with more robust performance, though differences in case difficulty between the RSNA Case Collection and Annual Meeting case sets may also contribute. Domain-specific factors and question format appear to influence success.

Our findings contrast with those of Hirosawa et al [22], who reported that GPT-4V attributed only 30% of decisions to image data on general case reports, with text-only GPT-4 outperforming GPT-4V (55.9% vs 44.4%). Our substantially higher image utilization (66%; 95% CI 63.5%-68.8%; Table S1 in Multimedia Appendix 1 and Table S5.5 in Multimedia Appendix 7) may reflect our explicit prompting to consider and quantify image information (Part A in Multimedia Appendix 4 documents modality quantification instructions) or the imaging-centric nature of neuroradiology cases compared to general medical case reports. These results suggest that GPT-4V makes extensive use of visual data, but improved outcomes depend on accurate image interpretation within the clinical context.

## Discussion

### Summary of Main Findings

This study addressed 2 primary objectives: evaluating GPT-4V’s diagnostic accuracy on expert-validated neuroradiology board-style questions and exploring self-reported reliance on imaging versus clinical text during diagnostic decision-making. Both objectives were successfully addressed. GPT-4V achieved 76% diagnostic accuracy on expert-validated neuroradiology cases, substantially exceeding prior performance on RSNA materials (76% vs 43% from Mukherjee et al [34] on case of the day challenges), though differences in case selection and difficulty limit direct comparison. Exploratory analysis revealed an inverse relationship: incorrect diagnoses were associated with higher self-reported visual reliance compared to correct diagnoses. While these represent the model’s self-characterization rather than validated measurements, this pattern generates testable hypotheses about multimodal integration failure modes. This is among the first studies systematically documenting how multimodal AI self-reports reliance on different information sources during clinical diagnosis.

### Interpretation and Comparison to Literature

Our findings contribute to emerging evidence regarding multimodal AI diagnostic accuracy and information integration patterns. Superior performance compared to prior studies [34] demonstrates that diagnostic accuracy depends critically on domain specificity, question format, and prompt engineering, all of which suggest that performance cannot be characterized by single global metrics but varies substantially based on the implementation approach.

The exploratory finding regarding modality attribution, where diagnostic errors were associated with higher self-reported image reliance, aligns with multiple studies. Schramm et al [20] identified textual descriptions as the strongest contributor to performance, while Hayden et al [16] found that GPT-4V performed significantly worse on image-based questions (47.8%) compared to text-only questions (81.5%). Albaqshi et al [17] demonstrated that image-only accuracy plummeted compared to text-with-image performance. Our pattern may reflect a failure mode in which the model attempts to extract diagnostic information primarily from visual data despite limited visual interpretation capabilities, thereby neglecting critical clinical context that might correct misinterpretations.

This image-dominant failure pattern warrants deeper mechanistic consideration. Incorrect diagnoses showed substantially higher self-reported image reliance compared to correct diagnoses, with a very large effect size, suggesting this is not merely statistical noise but a consistent pattern in self-reported attribution that determines whether this reflects actual information processing or post hoc rationalization. The fact that all incorrect diagnoses demonstrated image-dominant attribution patterns is particularly striking and suggests a potential “tipping point” beyond which diagnostic accuracy deteriorates markedly.

Several mechanisms could explain this pattern, though experimental validation is required to test these hypotheses. First, if visual processing capabilities lack domain-specific fine-grained discrimination for subtle radiological findings, excessive reliance on visual inputs might lead to confident but incorrect diagnoses. Second, the insufficient integration of clinical context could contribute to diagnostic errors. Third, architectural factors in multimodal integration remain unexplored and warrant investigation through controlled experiments with systematic input manipulation. These proposed mechanisms are speculative and require rigorous testing through image-only, text-only, and combined conditions to validate whether self-reported attribution patterns reflect actual information processing.

Comparison with human diagnostic patterns is instructive. Experienced radiologists typically use iterative hypothesis refinement, beginning with clinical context to generate differential diagnoses, and then using imaging to confirm or refute specific possibilities [30]. This approach naturally balances modalities by forcing explicit integration. In contrast, GPT-4V may process visual and textual streams more independently, with final outputs reflecting whichever stream activates more strongly rather than true synthesis. Potential architectural or integration limitation could explain why adding visual capabilities sometimes degrades rather than enhances performance [16,22,23], though our observational data cannot establish this mechanism.

Our finding of higher overall image utilization compared to some studies [22] may reflect explicit prompting to quantify image contribution or the imaging-centric nature of neuroradiology cases. However, the consistency of the image-dominant failure pattern across diverse pathology categories within neuroradiology (Table S6.2 in Multimedia Appendix 3) suggests this is not merely an artifact of case selection within this domain. Whether this pattern generalizes to other radiological subspecialties or medical domains requires investigation in diverse clinical contexts. Our observed overall image utilization, while seemingly reasonable, may actually be excessive given that textual clinical information often carries disproportionate diagnostic weight relative to its volume.

That Busch et al [21] demonstrated GPT-4V’s superiority over text-only approaches in some tasks suggests the relationship between modality contribution and diagnostic success is task-dependent and complex. This task dependency likely reflects varying degrees of diagnostic specificity achievable through visual inspection alone versus requiring clinical correlation. For conditions with pathognomonic imaging features (eg, calcified subependymal nodules in tuberous sclerosis), visual dominance may succeed. For conditions requiring clinical-radiological synthesis (eg, distinguishing demyelination patterns based on temporal profile), balanced integration becomes essential. Our results suggest that GPT-4V may not appropriately adjust modality weighting for different diagnostic scenarios, though whether this reflects limitations in actual processing versus self-assessment requires validation through controlled experiments.

The broader implications extend to fundamental questions about multimodal AI architecture. Current vision-language models typically use late fusion, where separate encoders process each modality before combining representations [35,36]. This approach, while computationally efficient, may fail to capture complex cross-modal dependencies essential for medical reasoning [37,38]. Early fusion architectures that enable deeper integration from initial processing stages, or attention mechanisms explicitly trained to modulate cross-modal influence based on task demands, may better support the dynamic modality balancing that expert diagnosis requires. Our finding that incorrect diagnoses systematically show imbalanced modality utilization provides empirical motivation for such architectural innovations.

### Clinical Safety and Deployment Implications

Examination of incorrect responses revealed 2 critical failure patterns that have distinct clinical implications. First, the model frequently generated hallucinated rationales citing nonexistent findings [24], consistent with documented hallucination rates of 35.5% to 63% in GPT-4V radiology applications [10,12,24]. Second, some errors reflected overemphasis on prominent visual findings while neglecting subtle clinical context, demonstrating that visual misinterpretations can lead the model astray when clinical information is insufficiently weighted.

These failure patterns carry distinct clinical risks requiring targeted mitigation strategies. Hallucinated findings are particularly dangerous because they appear authoritative and specific, potentially misleading clinicians who may not independently verify each claimed observation. In our study, hallucinations included references to imaging features not present in the provided images, incorrect anatomical localizations, and fabricated quantitative measurements. Such errors could lead to unnecessary interventions, incorrect diagnoses being entered into medical records, or delayed recognition of actual pathology.

The image-dominant failure mode presents a different risk profile. By over-weighting visual information that it cannot accurately interpret, GPT-4V may generate diagnoses that superficially align with prominent imaging features while missing the correct diagnosis that clinical context would suggest. This pattern is especially concerning in cases where imaging findings are nonspecific, but clinical history is highly discriminating. For example, ring-enhancing lesions have broad differential diagnoses, but patient age, immune status, and geographic location dramatically narrow possibilities [39]. A system that overrelies on imaging might suggest common etiologies based on visual appearance while missing the correct diagnosis apparent from clinical context.

These limitations mandate restricted deployment. GPT-4V should be implemented only as an educational tool or decision-support aid that highlights findings for human review but never as an autonomous diagnostic system. Any radiological application must include mandatory radiologist oversight, with AI output supplementing rather than replacing expert, as emphasized in multisociety professional guidelines [40-42]. Institutional protocols should explicitly prohibit applications bypassing human review. These restrictions remain necessary until multimodal integration capabilities achieve consistent, balanced utilization of both clinical and imaging information.

Specific implementation guidelines should include (1) interface design that presents AI outputs as preliminary suggestions explicitly requiring verification rather than definitive conclusions [43-45]; (2) transparent uncertainty quantification, ideally displaying the model’s self-reported modality contributions alongside confidence estimates to flag high-risk image-dominant attributions; (3) training programs educating users about characteristic failure modes, particularly the tendency toward hallucinated findings and image-dominant errors; (4) future quality assurance protocols could explore whether AI attribution patterns predict diagnostic errors, though the 70% threshold observed in our small sample requires validation across larger, diverse datasets before clinical implementation; and (5) mandatory documentation of AI involvement in clinical reports to ensure appropriate medicolegal clarity and enable post hoc analysis of AI-associated diagnostic errors.

Workflow integration must preserve rather than undermine human expertise. Systems should be designed as “AI-assisted” rather than “AI-augmented” workflows, maintaining radiologist agency and encouraging critical evaluation. Evidence from other domains suggests that over-reliance on AI recommendations (automation bias) can degrade human performance, particularly when users lack mechanisms to assess AI reliability [43,44]. Interfaces should therefore facilitate the easy verification of AI claims, such as by highlighting specific image regions purportedly showing claimed findings, enabling radiologists to quickly confirm or refute visual interpretations.

Regulatory frameworks must evolve to address multimodal AI’s unique challenges. Traditional medical device regulations focus on performance metrics including sensitivity, specificity, and accuracy but may inadequately address systematic failure modes, such as modality-specific overreliance or hallucination propensity [46,47]. Regulatory approval should require (1) comprehensive characterization of failure modes across diverse clinical scenarios, (2) validation that modality integration patterns align with domain expertise, (3) demonstration of appropriate uncertainty quantification, and (4) postmarket surveillance systems tracking AI-associated diagnostic errors. Our finding that image-dominant attribution predicts errors suggests that regulatory frameworks should incorporate modality balance metrics, potentially flagging deployments where typical attribution patterns diverge substantially from expert norms.

Educational implications are equally important. Radiology trainees must develop critical AI literacy, understanding both capabilities and characteristic failure modes of multimodal systems [48-50]. Training should include (1) recognition of hallucinated findings and strategies for systematic verification; (2) awareness that confident AI outputs may reflect overreliance on misinterpreted visual features; (3) skills in integrating AI suggestions with clinical reasoning rather than accepting them uncritically; and (4) understanding of when AI assistance is likely beneficial versus potentially misleading. Paradoxically, effective AI integration may require heightened rather than reduced emphasis on foundational clinical-radiological correlation skills [51-53], as overreliance on AI tools can diminish core competencies including diagnostic reasoning and clinical pattern recognition.

Comparison with human diagnostic errors provides important context. Radiologists also commit errors, with estimated miss rates varying by modality and pathology but often ranging from 3% to 5% for routine interpretations to 30% for subtle or complex findings [54,55]. However, human errors typically differ qualitatively from AI failures. Radiologists rarely hallucinate findings that do not exist; rather, they may overlook subtle abnormalities or misclassify ambiguous features [54,55]. Human errors often reflect attention limitations, cognitive biases, or knowledge gaps [56,57]. These are failure modes with well-established mitigation strategies, such as double-reading, checklists, and continuing education [58,59]. In contrast, AI hallucinations and systematic modality imbalances represent novel failure modes requiring new quality assurance approaches.

Our observed accuracy, while exceeding prior GPT-4V studies, remains below expert radiologist performance and insufficient for autonomous deployment. However, the more fundamental concern is not the accuracy level per se but the nature of failures. A system with 76% accuracy that fails randomly might be safely deployable with appropriate oversight, as human review would catch diverse errors. But a system showing systematic failure patterns (like our finding that image-dominant attribution reliably predicts errors) requires more cautious implementation, as certain case types may be systematically mishandled. Future deployment decisions must consider not only overall performance but failure pattern predictability and their alignment with human error patterns.

Our finding of higher overall image utilization compared to some studies [22] may reflect explicit prompting to quantify image contribution or the imaging-centric nature of neuroradiology cases. That Busch et al [21] demonstrated GPT-4V’s superiority over text-only approaches in some tasks suggests the relationship between modality contribution and diagnostic success is task-dependent and complex.

### Limitations

Several important limitations affect the interpretation of our findings. The primary concern is reliance on self-reported attribution of image versus text utilization, which may represent post hoc rationalizations rather than actual information processing. Rigorous validation requires controlled experiments comparing text-only, image-only, and multimodal conditions with information-theoretic metrics, such as mutual information between modalities and diagnostic accuracy.

The sample size of 29 neuroradiology cases limits statistical power for subgroup analyses and restricts generalizability to other radiological subspecialties. Performance in other subspecialties may differ substantially [21], and findings should not be extrapolated beyond adult neuroradiology. Multiple-choice format may overestimate performance relative to free-response clinical scenarios. The absence of ablation controls (text-only or image-only conditions) prevents the quantitative decomposition of relative modality contributions. Despite restricted RSNA access, data leakage cannot be definitively excluded with closed-source models. Finally, narrative justifications may not accurately reflect actual reasoning processes [24], limiting confidence in interpreting self-reported modality attribution.

### Implications

For AI in radiology, these results highlight the importance of moving beyond simple accuracy metrics toward mechanistic understanding of how multimodal systems process heterogeneous data. Future research should prioritize controlled experimental validation through systematic input manipulation, development of information-theoretic frameworks for quantifying true (rather than self-reported) modality contributions, and standardized test sets with confirmed provenance postdating model training to definitively address data leakage concerns.

Technical improvements must focus on enhancing multimodal integration through architectural innovations or specialized training that forces explicit cross-referencing of visual and textual features. Interface design should evolve beyond simply adding AI outputs to workflows; instead, it enables systems to express uncertainty transparently, highlight specific image regions, and respond to targeted clinician queries. Domain specialization through fine-tuning on curated radiology datasets remains essential, as general-purpose models exhibit variable performance across subspecialties.

Most critically, broader implications extend to establishing evidence-based frameworks for human-AI collaboration in clinical medicine. Current multimodal AI systems show promise as educational tools and decision-support aids but remain inappropriate for autonomous diagnostic applications. The field must resist premature deployment driven by technological enthusiasm, instead insisting on rigorous validation of both diagnostic accuracy and decision-making transparency. With continued technological advancement focused on balanced, context-aware data integration and systematic evaluation methodologies, future generations of multimodal AI may achieve robust, reliable performance necessary for meaningful contribution to radiologic practice and patient care.

### Conclusions: Broader Implications

This study contributes benchmark performance data and generates testable hypotheses about information integration patterns in diagnostic reasoning. The findings underscore that achieving high diagnostic accuracy requires more than adding visual capabilities to language models but demands sophisticated, balanced integration of clinical context and imaging findings. The exploratory observation that diagnostic failures may associate with imbalanced modality utilization suggests specific failure modes worthy of rigorous experimental investigation.

GPT-4V achieved 76% diagnostic accuracy on expert-validated neuroradiology cases, substantially exceeding prior GPT-4V performance on RSNA materials (43% by Mukherjee et al [34]). This improvement suggests that focused domain application with structured prompting may enhance performance, though experimental studies with controlled manipulation of these factors would be needed to establish causal relationships. However, the novel finding that all incorrect diagnoses associated with image-dominant attribution patterns, with substantially higher visual reliance than correct diagnoses and a very large effect size, identifies a potentially systematic failure mode requiring targeted mitigation. Until multimodal AI systems demonstrate consistent, balanced integration of clinical and imaging information with transparent uncertainty quantification, deployment should remain restricted to supervised educational and decision-support applications with mandatory radiologist oversight.

With continued technological advancement focused on balanced, context-aware data integration and systematic evaluation methodologies, future generations of multimodal AI may achieve the robust, reliable performance necessary for meaningful contribution to radiologic practice and patient care.

## Supplementary material

10.2196/69708 Multimedia Appendix 1 Diagnostic performance and self-reported modality attribution of GPT-4 with Vision in neuroradiology cases.

10.2196/69708 Multimedia Appendix 2 Study flow diagram and case distribution by pathology category for cross-sectional evaluation of GPT-4 with Vision diagnostic performance in neuroradiology.

10.2196/69708 Multimedia Appendix 3 Complete case catalog with metadata, modality attribution, and performance summary by pathology category.

10.2196/69708 Multimedia Appendix 4 Complete prompt template used to elicit diagnostic responses and modality attribution from GPT-4 with Vision, with representative example response.

10.2196/69708 Multimedia Appendix 5 Example of neuroradiology case questions for GPT-4 with Vision evaluation.

10.2196/69708 Multimedia Appendix 6 Operational definitions, measurement specifications, and validation rules for primary and exploratory variables in GPT-4 with Vision neuroradiology diagnostic study.

10.2196/69708 Multimedia Appendix 7 Data quality verification, statistical assumptions testing, and comprehensive descriptive statistics, including completeness analysis, outlier detection, normality assessment, and variance homogeneity for GPT-4 with Vision neuroradiology study.

10.2196/69708 Multimedia Appendix 8 Post hoc power analysis, sensitivity analysis, and sample size justification for cross-sectional study of GPT-4 with Vision diagnostic performance in neuroradiology.

10.2196/69708 Multimedia Appendix 9 Complete statistical analysis results including primary diagnostic accuracy, exploratory modality attribution comparisons, and distribution patterns for GPT-4 with Vision neuroradiology evaluation.

## Abbreviations

AI: artificial intelligence
CT: computed tomography
GPT-4V: GPT-4 with Vision
LLM: large language model
MRI: magnetic resonance imaging
RSNA: Radiological Society of North America
