# Supplementary material for: Diagnostic Accuracy of GPT-4 With Vision in Neuroradiology Board-Style Exam Questions: Cross-Sectional Case-Based Study
Source: JMIR Neurotechnol. 2026 Apr 30;5:e69708. doi: 10.2196/69708 (PMC13132487; doi:10.2196/69708)
Supplement: Multimedia Appendix 1 [file neuro-v5-e69708-s001.docx]

Multimedia Appendix 1: Diagnostic Performance and Self-Reported Modality Attribution of GPT-4V in Neuroradiology Cases

Table S1. Diagnostic Performance and Modality Attribution Analysis (N=29)

| Category | Image Attribution % (SD) [95% CI] | Text Attribution % (SD) [95% CI] | *P* Value |
| --- | --- | --- | --- |
| Overall Performance |  |  |  |
| Diagnostic accuracy | 22/29 correct (76%) [56–89] | — | <.001ᵃ |
|  |  |  |  |
| Modality Attribution |  |  |  |
| All cases | 66.1 (6.9) [63.5–68.8] | 33.9 (6.9) [31.2–36.5] | — |
| Correct diagnoses (n=22) | 62.8 (3.5) [61.3–64.3] | 37.2 (3.5) [35.7–38.7] | — |
| Incorrect diagnoses (n=7) | 76.7 (3.5) [73.5–79.9] | 23.3 (3.5) [20.1–26.5] | — |
|  |  |  |  |
| Group Comparison |  |  |  |
| Difference (incorrect − correct) | +13.9 percentage points | −13.9 percentage points | <.001ᵇ |
| Effect size (Cohen's *d*) | 4.08 | — | — |

ᵃ Binomial test vs 25% chance (z = 6.33). Wilson score method for CI.

ᵇ Two-sample *t*-test (*t*₂₇ = 9.15). Power >99.9%.

Note: Modality attribution reflects GPT-4V's self-reported reliance on visual versus textual information.

Diagnostic accuracy and self-reported information utilization patterns from cross-sectional evaluation of GPT-4 with Vision (GPT-4V) on 29 adult brain and central nervous system pathology cases from the Radiological Society of North America (RSNA) Case Collection (July 2024). Cases were authored by board-certified radiologists and included computed tomography (CT) and magnetic resonance imaging (MRI) studies with clinical vignettes, presented as four-option multiple-choice questions. GPT-4V achieved 76% diagnostic accuracy (22/29 cases correct; 95% CI: 56-89%), significantly exceeding chance performance (P<.001). Self-reported modality contributions showed overall mean image reliance of 66.1% and text reliance of 33.9%. Stratified by diagnostic outcome, correct diagnoses (n=22) demonstrated lower image reliance (62.8%) compared to incorrect diagnoses (n=7, 76.7%), with a mean difference of 13.9 percentage points (P<.001; Cohen's d=4.08). Values represent means ± standard deviation with 95% confidence intervals. Complete statistical methods detailed in Multimedia Appendices 4-9.
