# Supplementary material for: Diagnostic Accuracy of GPT-4 With Vision in Neuroradiology Board-Style Exam Questions: Cross-Sectional Case-Based Study
Source: JMIR Neurotechnol. 2026 Apr 30;5:e69708. doi: 10.2196/69708 (PMC13132487; doi:10.2196/69708)
Supplement: Multimedia Appendix 2 [file neuro-v5-e69708-s002.docx]

Multimedia Appendix 2: Study Flow Diagram and Case Distribution by Pathology Category for Cross-Sectional Evaluation of GPT-4V Diagnostic Performance in Neuroradiology

┌───────────────────────────────────────────────────────────┐

│ RSNA CASE COLLECTION SCREENING │

│ Total Available: 1,353 cases │

│ Neuroradiology Cases Reviewed: 289 │

│ Selection: First 29 Adult Brain/CNS Cases (July 2024) │

└─────────────────────────┬─────────────────────────────────┘

│

▼

┌────────────────────────────────────┐

│ INCLUSION CRITERIA │

│ • Age ≥18 years │

│ • Brain/CNS pathology only │

│ • CT or MRI imaging available │

│ • Multiple-choice format │

│ • Expert-verified diagnosis │

│ • Complete imaging studies │

└────────────────┬───────────────────┘

│

┌────────────────┴───────────────────┐

│ │

▼ ▼

┌────────────────┐ ┌──────────────────┐

│ EXCLUDED │ │ INCLUDED │

│ │ │ n = 29 │

│ • Pediatric │ │ │

│ • Non-brain/ │ │ • Publication: │

│ CNS │ │ 2020-2023 │

│ • Incomplete │ │ • 8 pathology │

│ imaging │ │ categories │

│ • Non-MC │ │ (Table S2.1) │

│ format │ │ │

└────────────────┘ └────────┬─────────┘

│

▼

┌─────────────────────────────┐

│ GPT-4V ASSESSMENT │

│ (July 2024) │

│ │

│ Platform: ChatGPT Plus │

│ Design: Single trial/case │

│ Prompt: Standardized │

│ Temperature: 1.0 │

│ Top-p: 1.0 │

│ │

│ All 29 cases assessed │

└──────────┬──────────────────┘

│

▼

┌─────────────────────────────┐

│ ANALYSIS (n=29) │

│ │

│ Data Completeness: 100% │

│ │

│ DIAGNOSTIC ACCURACY: │

│ • Correct: 22 (76%) │

│ • Incorrect: 7 (24%) │

│ │

│ MODALITY ATTRIBUTION: │

│ • Image: 66.1% │

│ • Text: 33.9% │

│ │

│ Missing Data: 0% │

└─────────────────────────────┘

# Figure S5.1. Flow Diagram for Cross-Sectional Study of GPT-4V Diagnostic Performance in Neuroradiology

Study flow from case selection through data analysis. From 1,353 total RSNA Case Collection cases, 289 neuroradiology cases were identified, and the first 29 adult brain and central nervous system pathology cases meeting inclusion criteria were selected in July 2024. All cases included complete CT or MRI imaging, clinical vignettes, and expert-verified multiple-choice diagnoses spanning 8 pathology categories (publication dates 2020-2023). GPT-4V assessment was conducted via ChatGPT Plus using standardized prompts (Appendix 1-Part A) with single-trial design and default parameters (temperature=1.0, top-p=1.0). Complete data were obtained for all 29 cases (100% completeness) with no missing values. GPT-4V achieved 76% diagnostic accuracy (22/29 correct) with self-reported mean modality contributions of 66.1% image and 33.9% text. CT, computed tomography; CNS, central nervous system; MC, multiple-choice; MRI, magnetic resonance imaging.

# Table S2.1. Case Distribution by Pathology Category

| Category | Cases | Correct | Incorrect |
| --- | --- | --- | --- |
| Vascular/Hemorrhagic | 5 | 5 | 0 |
| Infectious | 5 | 3 | 2 |
| Neoplastic | 4 | 3 | 1 |
| Metabolic/Toxic | 6 | 3 | 3 |
| Developmental/Congenital | 6 | 6 | 0 |
| Demyelinating | 1 | 1 | 0 |
| Traumatic | 1 | 1 | 0 |
| Genetic | 1 | 0 | 1 |

Note: Categories with single cases are shown for completeness. Sample sizes preclude statistical inference about category-specific performance.
