# Supplementary material for: Diagnostic Accuracy of GPT-4 With Vision in Neuroradiology Board-Style Exam Questions: Cross-Sectional Case-Based Study
Source: JMIR Neurotechnol. 2026 Apr 30;5:e69708. doi: 10.2196/69708 (PMC13132487; doi:10.2196/69708)
Supplement: Multimedia Appendix 3 [file neuro-v5-e69708-s003.docx]

Multimedia Appendix 3: Complete Case Catalog with Metadata, Modality Attribution, and Performance Summary by Pathology Category

# Table S6.1. Complete Study Sample: All 29 Cases with Metadata

| # | Pub Date | Pathology/Diagnosis | Category | Correct? | Image% | Text% | Notes |
| --- | --- | --- | --- | --- | --- | --- | --- |
| 1 | 11/15/2021 | Ruptured Spinal Dermoid | Congenital | ✓ | 60 | 40 | Balanced attribution |
| 2 | 4/19/2020 | Sturge-Weber Syndrome | Vascular | ✓ | 58 | 42 | Highest text reliance (correct) |
| 3 | 11/19/2020 | Cerebellar Extraventricular Neurocytoma | Neoplastic | ✓ | 65 | 35 | — |
| 4 | 3/21/2023 | Arachnoid Cyst | Congenital | ✓ | 67 | 33 | — |
| 5 | 8/9/2021 | Central Pontine Myelinolysis | Metabolic | ✓ | 61 | 39 | — |
| 6 | 7/22/2021 | CNS Capillary Telangiectasia | Vascular | ✓ | 59 | 41 | — |
| 7 | 12/14/2022 | Medulloblastoma | Neoplastic | ✓ | 66 | 34 | — |
| 8 | 12/1/2021 | Tuberculous Meningitis | Infectious | ✓ | 68 | 32 | — |
| 9 | 8/16/2021 | Developmental Venous Anomaly | Vascular | ✓ | 61 | 39 | — |
| 10 | 11/1/2021 | Dural Arteriovenous Fistula | Vascular | ✓ | 60 | 40 | — |
| 11 | 6/21/2021 | Meningeal Neurosarcoidosis | Inflammatory | ✓ | 57 | 43 | Lowest image reliance (overall) |
| 12 | 10/14/2020 | Pilocytic Astrocytoma | Neoplastic | ✓ | 70 | 30 | Image-dominant but correct |
| 13 | 5/28/2020 | Jugular Bulb Diverticulum | Anatomic Variant | ✓ | 63 | 37 | — |
| 14 | 8/10/2022 | Neurocysticercosis | Infectious | ✓ | 65 | 35 | Example case (Fig 2) |
| 15 | 5/15/2023 | Osmotic Demyelination | Metabolic | ✓ | 61 | 39 | — |
| 16 | 4/4/2023 | Leptomeningeal Siderosis | Hemorrhagic | ✓ | 59 | 41 | — |
| 17 | 2/21/2022 | Tumefactive Multiple Sclerosis | Demyelinating | ✓ | 64 | 36 | — |
| 18 | 8/9/2021 | Vertex Epidural Hematoma | Traumatic | ✓ | 66 | 34 | — |
| 19 | 11/27/2020 | Syntelencephaly | Developmental | ✓ | 63 | 37 | Example case (Fig 3) |
| 20 | 7/7/2021 | Hypernatremic Osmotic Demyelination | Metabolic | ✓ | 62 | 38 | — |
| 21 | 3/6/2023 | Enlarged Parietal Foramina | Congenital | ✓ | 62 | 38 | — |
| 22 | 1/22/2021 | Filum Terminale Lipoma | Congenital | ✓ | 64 | 36 | — |
|  |  |  |  |  |  |  |  |
|  | CORRECT SUBTOTAL |  |  | 22 | M=62.8 | M=37 | SD=3.39 |
| 23 | 4/20/2020 | Fahr's Syndrome | Metabolic | ✗ | 74 | 26 | Lowest image% (incorrect) |
| 24 | 3/6/2023 | Tuberculous Leptomeningitis | Infectious | ✗ | 80 | 20 | Outlier (high image%) |
| 25 | 6/7/2021 | Mitochondrial Encephalopathy | Metabolic | ✗ | 82 | 18 | Highest image reliance (overall) |
| 26 | 5/5/2020 | Acute Bilirubin Encephalopathy | Metabolic | ✗ | 78 | 22 | — |
| 27 | 2/17/2021 | Myxopapillary Ependymoma | Neoplastic | ✗ | 72 | 28 | Lowest image% (incorrect) |
| 28 | 3/10/2022 | CNS Tuberculosis | Infectious | ✗ | 75 | 25 | — |
| 29 | 10/11/2021 | Lipoid Proteinosis | Genetic | ✗ | 76 | 24 | — |
|  | INCORRECT SUBTOTAL |  |  | 7 | M=76.7 | M=23.3 | SD=3.50 |
|  | TOTAL |  |  | 29 | M=66.1 | M=33.86 | SD=6.94 |

## Table S6.2. Performance Summary by Pathology Category

| Category | Cases | Correct | Incorrect | Accuracy | Mean Image% (All) | Mean Image% (Correct) | Mean Image% (Incorrect) |
| --- | --- | --- | --- | --- | --- | --- | --- |
| Vascular/Hemorrhagic | 5 | 5 | 0 | 100% | 59.6 | 59.6 | — |
| Developmental/Congenital | 6 | 6 | 0 | 100% | 63.2 | 63.2 | — |
| Demyelinating | 1 | 1 | 0 | 100% | 64.0 | 64.0 | — |
| Traumatic | 1 | 1 | 0 | 100% | 66.0 | 66.0 | — |
| Neoplastic | 4 | 3 | 1 | 75% | 68.3 | 67.0 | 72.0 |
| Infectious/Inflammatory | 5 | 3 | 2 | 60% | 68.8 | 63.3 | 77.5 |
| Metabolic/Toxic | 6 | 3 | 3 | 50% | 69.7 | 61.3 | 78.0 |
| Genetic | 1 | 0 | 1 | 0% | 76.0 | — | 76.0 |
