# Supplementary material for: Diagnostic Accuracy of GPT-4 With Vision in Neuroradiology Board-Style Exam Questions: Cross-Sectional Case-Based Study
Source: JMIR Neurotechnol. 2026 Apr 30;5:e69708. doi: 10.2196/69708 (PMC13132487; doi:10.2196/69708)
Supplement: Multimedia Appendix 4 [file neuro-v5-e69708-s004.docx]

Multimedia Appendix 4: Complete Prompt Template Used to Elicit Diagnostic Responses and Modality Attribution from GPT-4V, with Representative Example Response

# Part A: Complete Prompt Structure

## Each case was presented to GPT-4V using the following standardized prompt template:

[CLINICAL VIGNETTE AND CASE INFORMATION]

Gender: [Male/Female]

Age: [XX years old]

Clinical History & Presentation:

[Full clinical vignette text as provided in RSNA case]

[UPLOADED IMAGES]

[All CT/MRI images for the case were uploaded as separate image files]

## QUESTION:

Based on the clinical history and the provided imaging, what is the most likely diagnosis?

ANSWER CHOICES:

A. [Option A]

B. [Option B]

C. [Option C]

D. [Option D]

## INSTRUCTIONS:

Please complete the following tasks in order:

- Commit to your answer first: State your final answer choice (A, B, C, or D) at the very beginning of your response, before providing any explanation.
- Provide your reasoning: Explain your diagnostic reasoning, describing the relevant imaging findings and clinical features that support your answer.
- Quantify information sources: Estimate the percentage contribution of the following information sources to your diagnostic decision:
- Image data: What percentage of your reasoning was based on the visual imaging findings you observed in the provided scans?
- Clinical text: What percentage of your reasoning was based on the patient history and clinical presentation provided in the text?
- Please report these percentages (which should sum to 100%) at the end of your response.
- Modality Attribution:
  - Image contribution: [X]%
  - Text contribution: [Y]%

# Part B: Representative Example Response from GPT-4V


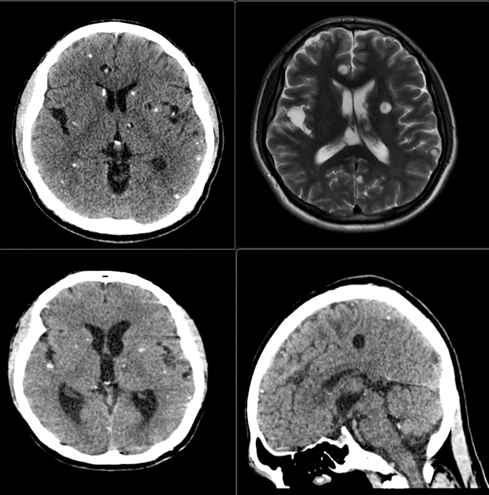


## Figure S2. Representative Infectious Disease Neuroradiology Case from RSNA Case Collection Demonstrating Clinical Context Integration in GPT-4V Multimodal Diagnostic Decision-Making

This figure illustrates a typical case-based question format used in the study to evaluate GPT-4V’s diagnostic accuracy. The case includes a clinical vignette describing a 32-year-old male presenting with a first-time seizure, along with relevant CT/MRI images. Four multiple-choice options representing potential diagnoses are provided. The model is prompted to review the imaging and clinical history before selecting its diagnosis, followed by an explanation of how much image and text data contributed to its answer.

Sample Question for Figure S2

Clinical History & Presentation:
A 32-year-old male with no significant past medical history presented to the emergency department (ED) after a witnessed tonic-clonic seizure. The episode began with a few seconds of dizziness and syncope, progressing to the seizure. The patient denied having any preceding aura or drug use. He was born in Guatemala and moved to the United States one year ago.

Question: Based on the clinical history and the provided imaging, what is the most likely diagnosis?

Answer Choices:

A. Neurocysticercosis

B. Brain abscesses

C. Central Nervous System Tuberculoma

D. Brain metastases

Images:
(CT/MRI images corresponding to the case showing relevant neurological findings)

GPT-4V Response:

Answer: A

Reasoning:

- The most likely diagnosis is neurocysticercosis.
- The imaging demonstrates multiple ring-enhancing lesions scattered throughout both cerebral hemispheres, with associated perilesional edema. These lesions show the characteristic "target sign" appearance with central hypodensity and peripheral enhancement, which is highly suggestive of cysticercal cysts in various stages of degeneration. The distribution pattern—multiple lesions at the gray-white matter junction—is classic for neurocysticercosis.
- The clinical history strongly supports this diagnosis. The patient is a 32-year-old male who immigrated from Guatemala one year ago. Guatemala is endemic for Taenia solium infection, making neurocysticercosis the most common cause of adult-onset seizures in immigrants from Latin America. The presentation with a first-time tonic-clonic seizure without prior aura is typical for symptomatic neurocysticercosis, where seizures result from the inflammatory response to dying parasites.
- While brain abscesses, tuberculomas, and metastases can produce similar ring-enhancing lesions, several features make these less likely:
- Brain abscesses typically present with fever and more significant surrounding edema; this patient has no mention of systemic infection signs
- CNS tuberculomas are less common in this demographic without known TB exposure
- Brain metastases are unlikely in a young patient without known primary malignancy
- The combination of the imaging pattern, epidemiological background, and clinical presentation makes neurocysticercosis the most probable diagnosis.

Modality Attribution:

- Image contribution: 65%
- Text contribution: 35%

# Part C: Notes on Response Variability

Across the 29 cases evaluated, GPT-4V's responses showed the following patterns:

- Format compliance: All 29 responses followed the requested format with answer-first structure and modality attribution percentages
- Attribution range: Self-reported image contribution ranged from 40% to 90%, with a mean of 67%
- Percentage precision: The model typically reported percentages in 5% increments (e.g., 60%/40%, 65%/35%, 70%/30%)
- Sum verification: All reported percentages correctly summed to 100%
- The model consistently provided the modality attribution at the end of its response in the requested format, facilitating systematic extraction and analysis.

# Part D: Data Extraction Process

## Extraction Methodology

For each of the 29 cases, data were extracted manually from GPT-4V's text responses within the ChatGPT interface. The extraction process was as follows:

Answer choice extraction: The letter answer (A, B, C, or D) was recorded from the first line of each response, where the model consistently stated "Answer: [X]" per the prompt instructions.

Modality attribution extraction: The percentage values were manually transcribed from the "Modality Attribution" section at the end of each response. The model consistently formatted these values as:

"Image contribution: [X]%"

"Text contribution: [Y]%"

These percentages were directly copied into a spreadsheet (Microsoft Excel) with columns labeled "Image_Percentage" and "Text_Percentage."

Verification checks: After initial extraction, all entries were double-checked to ensure:

- No transcription errors occurred during manual entry.
- The answer choice matched the model's stated selection.

Correctness determination: Each answer choice was compared against the expert-established correct answer from the RSNA case documentation. Cases were coded as correct (1) or incorrect (0) in a binary "Correctness" column.

Qualitative reasoning capture: The full text of the model's reasoning explanation was copied into a separate column for subsequent qualitative analysis of error patterns.

## Data Format

The extracted data were organized in spreadsheet format with the following structure:

| Case_ID (Pub Date) | Pathology/  Topic | Correct | Image_% | Text_% | Reasoning Text |
| --- | --- | --- | --- | --- | --- |
| 11/15/2021 | Ruptured Spinal Dermoid | Correct | 60 | 40 | The sagittal T1-weighted post-contrast MRI shows a well-defined, enhancing intradural extramedullary mass in the conus medullaris / filum terminale region. The lesion demonstrates moderate enhancement and expands the distal thecal sac — classic for myxopapillary ependymoma, the most common tumor of this location in young adults. |
| ... |  |  |  |  |  |
| ... | ... | ... | ... | ... | ... |

No Automated Parsing.

We note that extraction was performed entirely manually rather than through automated text parsing or API-based data retrieval. This approach was chosen because:

- The ChatGPT Plus web interface does not provide programmatic API access with structured output.
- Manual extraction allowed verification of response quality and format compliance.
- With only 29 cases, manual extraction was feasible and allowed immediate identification of any anomalous responses.

The manual extraction process was completed by [Author Initials], with verification performed by [Author Initials] to ensure accuracy. No discrepancies were identified during the verification process.

# Part E: Reproducibility Statement

This appendix provides complete documentation of the prompt template and expected response format to enable exact replication of our methodology. Researchers seeking to reproduce or extend this work can use the identical prompt structure, instructions, and response format specifications documented here.
