# Supplementary material for: Diagnostic Accuracy of GPT-4 With Vision in Neuroradiology Board-Style Exam Questions: Cross-Sectional Case-Based Study
Source: JMIR Neurotechnol. 2026 Apr 30;5:e69708. doi: 10.2196/69708 (PMC13132487; doi:10.2196/69708)
Supplement: Multimedia Appendix 5 [file neuro-v5-e69708-s005.docx]

Multimedia Appendix 5: Example Neuroradiology Case Questions for GPT-4V Evaluation

# Figure S3 with complete case presentations and imaging examples from the RSNA Case Collection.

All cases underwent peer review and editorial vetting by RSNA's editorial board prior to publication [Official RSNA Case Collection Author Guidelines (PDF) available from the RSNA Case Collection website’s assets: cases.rsna.org/assets/documents/authoring_instructions.pdf].


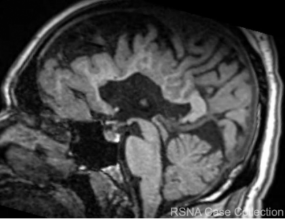

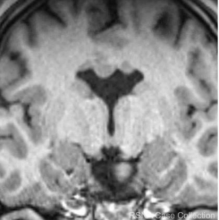

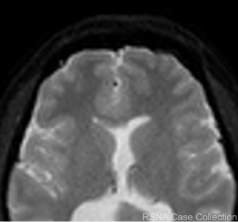

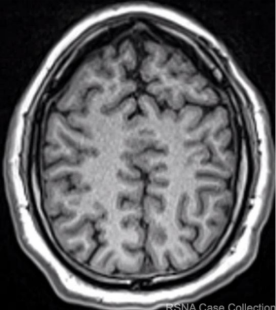

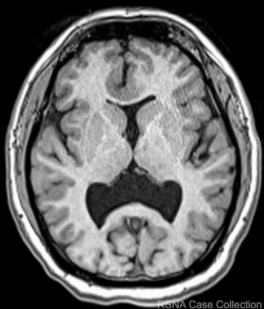


## Figure S3. Representative Developmental Brain Malformation Case Highlighting Complex Multimodal Integration Requirements for Accurate AI Diagnosis

This figure demonstrates a complex neuroradiology case that requires both clinical history and imaging analysis for accurate diagnosis. The case involves a patient with developmental abnormalities and seizures, where the diagnosis depends heavily on recognizing specific imaging patterns in conjunction with the clinical presentation. This example emphasizes the necessity of multimodal integration for successful AI diagnosis in challenging neuroradiology cases.

### Sample Question for Figure S3

Clinical History & Presentation:
A 31-year-old male patient presents with a history of prematurity, lifetime intellectual impairment, and seizure episodes. Patient had delayed motor and speech development after the first year of life. Patient attends an adult daycare program. Seizure episodes include spells of staring/smiling and bilateral repetitive hand movements. No known prior genetic testing or prenatal imaging has been performed.

Question: Based on the clinical history and the provided imaging, what is the most likely diagnosis?

Answer Choices:

A. Syntelencephaly (Middle Interhemispheric Variant)

B. Schizencephaly

C. Lobar Holoprosencephaly

D. Septo-Optic Dysplasia

Images: (MRI images corresponding to the case showing developmental brain abnormalities)

Correct Answer: Syntelencephaly (Middle Interhemispheric Variant of Holoprosencephaly)
