# Supplementary material for: Diagnostic Accuracy of GPT-4 With Vision in Neuroradiology Board-Style Exam Questions: Cross-Sectional Case-Based Study
Source: JMIR Neurotechnol. 2026 Apr 30;5:e69708. doi: 10.2196/69708 (PMC13132487; doi:10.2196/69708)
Supplement: Multimedia Appendix 6 [file neuro-v5-e69708-s006.docx]

Multimedia Appendix 6: Operational Definitions, Measurement Specifications, and Validation Rules for Primary and Exploratory Variables in GPT-4V Neuroradiology Diagnostic Study

# Table S7.1. Primary and Exploratory Variables

| Variable Name | Type | Range/Values | Operational Definition | Measurement Level |
| --- | --- | --- | --- | --- |
| pub_date | Date | 4/19/2020 to 5/15/2023 | RSNA case publication date | Ordinal |
| pathology | Text | 29 unique diagnoses | Primary diagnosis from RSNA case title | Nominal |
| correct | Binary | 0, 1 | Diagnostic accuracy: 1 if GPT-4V answer matches RSNA correct answer; 0 otherwise | Nominal |
| image_pct | Continuous | 57-82 | Self-reported percentage (0-100) of diagnostic decision attributed to imaging data | Ratio |
| text_pct | Continuous | 18-43 | Self-reported percentage (0-100) of diagnostic decision attributed to clinical text | Ratio |

# Table S7.2. Data Validation Rules Applied

| Validation Check | Rule | Pass Criteria | Result |
| --- | --- | --- | --- |
| No missing values | All cells populated | 0% missing | 100% complete |
| Binary coding | correct ∈ {0, 1} | Only 0 or 1 | All valid |
| Percentage sum | image_pct + text_pct = 100 | All sum to 100% | 29/29 verified |
| Range validity | image_pct ∈ [0, 100] | Within bounds | [57, 82] valid |
| Range validity | text_pct ∈ [0, 100] | Within bounds | [18, 43] valid |
| Response format | Letter A/B/C/D only | Single letter | 29/29 compliant |
| Unique cases | No duplicates | Unique pub_date + pathology | All unique |

# 8.1 Detailed Variable Specifications

VARIABLE: correct (Primary Outcome)

Definition: Binary indicator of whether GPT-4V selected the RSNA-verified correct diagnosis

Coding Rules:

1 (Correct): GPT-4V's selected answer (A, B, C, or D) exactly matches RSNA correct answer

0 (Incorrect): GPT-4V's selected answer does not match RSNA correct answer

No partial credit given

Missing or ambiguous responses would be coded as 0 (did not occur: n=0)

Reference Standard: RSNA Case Collection expert-verified correct answer, determined by:

Board-certified radiologist case author

RSNA editorial peer review

Published answer key in case documentation

Extraction Method: Answer letter extracted from first line of GPT-4V response ("Answer: [X]")

Aggregate Metric: Diagnostic accuracy = (Σ correct) / 29 × 100%

VARIABLE: image_pct (Exploratory Outcome)

Definition: GPT-4V's self-reported percentage contribution of imaging/visual data to its diagnostic decision

Elicitation Method: Obtained via standardized prompt requesting percentage quantification:

"Estimate the percentage contribution... Image data: What percentage of your reasoning was based on the visual imaging findings you observed in the provided scans?"

Format: Integer percentages (whole numbers)

Model typically reported in 5% increments (60%, 65%, 70%)

Range: 57% to 82%

Mean: 66.14% (SD=6.97)

Mathematical Constraint: image_pct + text_pct = 100% (verified for all cases)

Interpretation Limitations:

Represents model's self-characterization, NOT validated measure of actual computational process

May reflect post-hoc rationalization rather than true information weighting

Requires experimental validation through controlled text-only and image-only conditions

Should be interpreted as exploratory, hypothesis-generating data

Extraction Method: Manually transcribed from "Image contribution: [X]%" in GPT-4V response

VARIABLE: text_pct (Exploratory Outcome)

Definition: GPT-4V's self-reported percentage contribution of clinical/textual data to its diagnostic decision

Relationship: Mathematically complementary to image_pct (text_pct = 100 - image_pct)

Same interpretation limitations as image_pct apply

# 8.2 Derived Variables for Analysis

| Derived Variable | Formula/Rule | Purpose |
| --- | --- | --- |
| outcome_group | IF correct=1 THEN "Correct" ELSE "Incorrect" | Grouping variable for t-test |
| text_image_ratio | text_pct / image_pct | Relative reliance metric (e.g., 0.59 = ~1.7:1 image-to-text) |
| image_dominant | IF image_pct ≥ 70 THEN 1 ELSE 0 | Binary indicator: high image reliance |
| balanced_attribution | IF image_pct BETWEEN 55 AND 69 THEN 1 ELSE 0 | Binary indicator: balanced integration |
| year | YEAR(pub_date) | Publication year (2020-2023) |
