# Supplementary material for: Diagnostic Accuracy of GPT-4 With Vision in Neuroradiology Board-Style Exam Questions: Cross-Sectional Case-Based Study
Source: JMIR Neurotechnol. 2026 Apr 30;5:e69708. doi: 10.2196/69708 (PMC13132487; doi:10.2196/69708)
Supplement: Multimedia Appendix 7 [file neuro-v5-e69708-s007.docx]

Multimedia Appendix 7: Data Quality Verification, Statistical Assumptions Testing, and Comprehensive Descriptive Statistics Including Completeness Analysis, Outlier Detection, Normality Assessment, and Variance Homogeneity for GPT-4V Neuroradiology Study

# Table S5.1. Data Completeness Verification

| Variable | Expected n | Obtained n | Completeness | Missing |
| --- | --- | --- | --- | --- |
| Publication Date | 29 | 29 | 100% | 0 |
| Pathology/Diagnosis | 29 | 29 | 100% | 0 |
| Diagnostic Response (A/B/C/D) | 29 | 29 | 100% | 0 |
| Correctness (Binary) | 29 | 29 | 100% | 0 |
| Image % Attribution | 29 | 29 | 100% | 0 |
| Text % Attribution | 29 | 29 | 100% | 0 |
| Reasoning Text | 29 | 29 | 100% | 0 |
| TOTAL | 203 data points | 203 | 100% | 0 |

# Table S5.2. Outlier Analysis (Tukey's Method, IQR × 1.5)

Image % Attribution - Overall Sample (n=29)

| Statistic | Correct Value |
| --- | --- |
| Mean | 66.14 |
| Median | 64.0 |
| Q1 (25th percentile) | 61.00 |
| Q3 (75th percentile) | 70.00 |
| IQR | 9.00 |
| Lower fence (Q1 - 1.5×IQR) | 47.50 |
| Upper fence (Q3 + 1.5×IQR) | 83.50 |
| Outliers identified | 0 cases |

# Table S5.3. Normality Assessment (Shapiro-Wilk Tests)

| Variable & Group | n | W statistic | *P-*value | Distribution |
| --- | --- | --- | --- | --- |
| Image% - All cases | 29 | 0.908 | .015 | Non-normal* |
| Image% - Correct only | 22 | 0.981 | .937 | Normal |
| Image% - Incorrect only | 7 | 0.978 | .947 | Normal |
| Text% - All cases | 29 | 0.908 | .015 | Non-normal* |
| Text% - Correct only | 22 | 0.981 | .937 | Normal |
| Text% - Incorrect only | 7 | 0.978 | .947 | Normal |

*Overall non-normality due to bimodal distribution (two distinct groups)

# Table S5.4. Homogeneity of Variance (Levene's Test)

## Test: Do correct and incorrect groups have equal variances?

| Statistic | Value |
| --- | --- |
| F-statistic | 0.003 |
| df₁ | 1 |
| df₂ | 27 |
| *P*-value | .957 |
| Conclusion | Equal variances assumed |

# Table S5.5. Summary Statistics - All Variables

## Summary Statistics - All Variables

| Variable | n | Mean | SD | Median | Min | Max | Range | Skewness | Kurtosis |
| --- | --- | --- | --- | --- | --- | --- | --- | --- | --- |
| Image% - All | 29 | 66.14 | 6.94 | 64.0 | 57 | 82 | 25 | 0.83 | -0.39 |
| Image% - Correct | 22 | 62.77 | 3.39 | 62.5 | 57 | 70 | 13 | 0.27 | -0.65 |
| Image% - Incorrect | 7 | 76.71 | 3.50 | 76.0 | 72 | 82 | 10 | 0.23 | -1.11 |
| Text% - All | 29 | 33.86 | 6.94 | 36.0 | 18 | 43 | 25 | -0.83 | -0.39 |
| Text% - Correct | 22 | 37.23 | 3.39 | 37.5 | 30 | 43 | 13 | -0.27 | -0.65 |
| Text% - Incorrect | 7 | 23.29 | 3.50 | 24.0 | 18 | 28 | 10 | -0.23 | -1.11 |
