# Supplementary material for: Diagnostic Accuracy of GPT-4 With Vision in Neuroradiology Board-Style Exam Questions: Cross-Sectional Case-Based Study
Source: JMIR Neurotechnol. 2026 Apr 30;5:e69708. doi: 10.2196/69708 (PMC13132487; doi:10.2196/69708)
Supplement: Multimedia Appendix 8 [file neuro-v5-e69708-s008.docx]

Multimedia Appendix 8: Post-Hoc Power Analysis, Sensitivity Analysis, and Sample Size Justification for Cross-Sectional Study of GPT-4V Diagnostic Performance in Neuroradiology

# Table S3.1. Post-Hoc Power Analysis Results

## Primary Analysis: Diagnostic Accuracy vs. Chance

| Parameter | Value |
| --- | --- |
| Sample size (n) | 29 |
| Observed accuracy | 75.9% (22/29) |
| Null hypothesis (chance) | 25% |
| Effect size (Cohen's h) | 1.07 |
| Alpha level | 0.05 (two-tailed) |
| Achieved power | >99.9% |
| Conclusion | Highly powered to detect observed effect |

## Exploratory Analysis: Modality Attribution by Outcome

| Parameter | Correct Diagnoses (n=22) | Incorrect Diagnoses (n=7) | Difference |
| --- | --- | --- | --- |
| Mean image attribution, % (SD) | 62.8 (3.4) | 76.7 (3.5) | 13.9 percentage points |
| Effect size (Cohen's d) | — | — | 4.08 |
| Statistical comparison | — | — | t(27) = 9.40, *P* <.001 |
| Achieved power, % | — | — | >99.9 |

## Sensitivity Analysis: Minimum Detectable Effects

| Effect Size | d | Power (n₁=22, n₂=7) | Sample Size for 80% Power |
| --- | --- | --- | --- |
| Small | 0.2 | 7% | n₁≥590, n₂≥295 or n=394/group |
| Medium | 0.5 | 20% | n₁≥96, n₂≥48 or n=64/group |
| Large | 0.8 | 43% | n₁≥39, n₂≥20 or n=26/group |
| Very Large | 1.76 | 97% | n₁≥20, n₂≥10 or n=7/group |

## Table S3.2. Precision Analysis for Diagnostic Accuracy

| Measure | Value | Method |
| --- | --- | --- |
| Observed accuracy | 75.9% (22/29) | Point estimate |
| 95% CI Lower Bound | 56.5% | Wilson score |
| 95% CI Upper Bound | 89.1% | Wilson score |
| CI Width | 32.6 percentage points | — |
| Margin of Error | ±16.3 percentage points | — |
| Excludes chance (25%)? | Yes | Lower CI > 25% |
