# Supplementary material for: Diagnostic Accuracy of GPT-4 With Vision in Neuroradiology Board-Style Exam Questions: Cross-Sectional Case-Based Study
Source: JMIR Neurotechnol. 2026 Apr 30;5:e69708. doi: 10.2196/69708 (PMC13132487; doi:10.2196/69708)
Supplement: Multimedia Appendix 9 [file neuro-v5-e69708-s009.docx]

Multimedia Appendix 9: Complete Statistical Analysis Results Including Primary Diagnostic Accuracy, Exploratory Modality Attribution Comparisons, and Distribution Patterns for GPT-4V Neuroradiology Evaluation

# Table S4.1. Primary Outcome: Diagnostic Accuracy

| Measure | Value | 95% Confidence Interval |
| --- | --- | --- |
| Total cases assessed | 29 | — |
| Correct diagnoses | 22 | — |
| Incorrect diagnoses | 7 | — |
| Diagnostic accuracy | 75.9% | 57.9%, 87.8%] |
| Expected under chance | 25.0% | — |
| Difference from chance | +50.9 percentage points | — |

Statistical Test: One-Sample Binomial Test

H₀: Accuracy = 25% (random guessing for 4 options)

H₁: Accuracy ≠ 25% (two-tailed)

Test statistic: z = 6.33

*P*-value: <.001

# Table S4.2. Exploratory Outcome: Modality Attribution by Diagnostic Outcome

## Descriptive Statistics

| Group | n | Mean Image% | SD | 95% CI | Median | Range |
| --- | --- | --- | --- | --- | --- | --- |
| Correct diagnoses | 22 | 62.77 |  | 3.39 [61.17, 64.37] | 63.0 | [57, 70] |
| Incorrect diagnoses | 7 | 76.71 | 3.50 | [73.68, 79.75] | 76.0 | [72, 82] |
| Difference | — | 13.94 | — | [10.38, 17.50] | — | — |

## Corresponding Text Attribution

| Group | n | Mean Text% | SD | 95% CI |
| --- | --- | --- | --- | --- |
| Correct diagnoses | 22 | 37.23 | 3.39 | [35.63, 38.83] |
| Incorrect diagnoses | 7 | 23.29 | 3.50 | [20.25, 26.32] |

## Text:Image Ratios

| Context | Ratio | Interpretation |
| --- | --- | --- |
| Overall | 0.51:1 | ~2:1 image-to-text reliance |
| Correct diagnoses | 0.59:1 | ~1.7:1 image-to-text |
| Incorrect diagnoses | 0.30:1 | ~3.3:1 image-to-text |

## Statistical Test: Independent Samples t-Test

| Parameter | Value |
| --- | --- |
| Hypotheses | H₀: μ_incorrect = μ_correct vs. H₁: μ_incorrect ≠ μ_correct |
| Mean difference | 13.94 percentage points (incorrect higher) |
| Standard error | 1.48 |
| t-statistic | 9.40 |
| Degrees of freedom | 27 |
| *P-*value | <.001 |
| 95% CI for difference | [10.38, 17.50] percentage points |

# Table S4.3. Distribution of Attribution Patterns

| Attribution Pattern | Criterion | Correct (n=22) | Incorrect (n=7) | Total |
| --- | --- | --- | --- | --- |
| Image-dominant | Image ≥ 70% | 1 (4.5%) | 7 (100%) |  |
| Balanced | Image 55-69% | 21 (95.5%) | 0 (0%) | 19 |
| Text-dominant | Image ≤ 54% | 0 (0%) | 0 (0%) | 0 |
